# An intrinsic agonist mechanism for activation of glucagon-like peptide-1 receptor by its extracellular domain

**DOI:** 10.1038/celldisc.2016.42

**Published:** 2016-11-22

**Authors:** Yanting Yin, X Edward Zhou, Li Hou, Li-Hua Zhao, Bo Liu, Gaihong Wang, Yi Jiang, Karsten Melcher, H Eric Xu

**Affiliations:** 1 Key Laboratory of Receptor Research, VARI-SIMM Center, Center for Structure and Function of Drug Targets, Shanghai Institute of Materia Medica, Chinese Academy of Sciences, Shanghai, China; 2 Laboratory of Structural Sciences and Laboratory of Structural Biology and Biochemistry, Van Andel Research Institute, Grand Rapids, MI, USA; 3 University of Chinese Academy of Sciences, Beijing, China

**Keywords:** GLP-1R, class B GPCR, intrinsic agonist, glucagon-like peptide-1, exendin-4, BETP

## Abstract

The glucagon-like peptide-1 receptor is a class B G protein coupled receptor (GPCR) that plays key roles in glucose metabolism and is a major therapeutic target for diabetes. The classic two-domain model for class B GPCR activation proposes that the apo-state receptor is auto-inhibited by its extracellular domain, which physically interacts with the transmembrane domain. The binding of the C-terminus of the peptide hormone to the extracellular domain allows the N-terminus of the hormone to insert into the transmembrane domain to induce receptor activation. In contrast to this model, here we demonstrate that glucagon-like peptide-1 receptor can be activated by N-terminally truncated glucagon-like peptide-1 or exendin-4 when fused to the receptor, raising the question regarding the role of N-terminal residues of peptide hormone in glucagon-like peptide-1 receptor activation. Mutations of cysteine 347 to lysine or arginine in intracellular loop 3 transform the receptor into a G protein-biased receptor and allow it to be activated by a nonspecific five-residue linker that is completely devoid of exendin-4 or glucagon-like peptide-1 sequence but still requires the presence of an intact extracellular domain. Moreover, the extracellular domain can activate the receptor in trans in the presence of an intact peptide hormone, and specific mutations in three extracellular loops abolished this extracellular domain trans-activation. Together, our data reveal a dominant role of the extracellular domain in glucagon-like peptide-1 receptor activation and support an intrinsic agonist model of the extracellular domain, in which peptide binding switches the receptor from the auto-inhibited state to the auto-activated state by releasing the intrinsic agonist activity of the extracellular domain.

## Introduction

The glucagon-like peptide-1 receptor (GLP-1R) [1] is a class B G protein coupled receptor (GPCR) whose endogenous agonist glucagon-like peptide-1 is a 30-amino acid peptide hormone. GLP-1R plays a central role in post-prandial insulin release [2]; the inhibition of gastric emptying [3, 4]; the inhibition of glucagon secretion [5] and the reduction of food intake [6], thus GLP-1R is an important pharmaceutical target for diabetes therapy. Another potent GLP-1R agonist is exendin-4 (EX4), a 39-amino acid natural analog of GLP-1 found in the saliva of the Gila monster [7, 8]. It is 50% identical to GLP-1 with a nine-residue C-terminal extension, and is of more potential pharmacological interest due to its higher binding affinity to GLP-1R [9]. EX4 has been developed as an anti-diabetic drug under the name of exenatide, which was licensed for use as an adjunct therapy [10, 11]. However, the disadvantage of a peptide drug is its short circulating plasma half-life and unfavorable parenteral drug delivery [12, 13]. Designing better anti-diabetic peptide drugs or small molecule analogs are the focus of many intensive pharmaceutical research efforts. A structural model of hormone binding and receptor activation, however, remains elusive.

Currently there are three published crystal structures of class B GPCR transmembrane domains (TMD) with stabilizing small molecular antagonists [14–16], and a dozen structures of extracellular domain (ECD)-peptide hormone complexes [17–23]. The TMD structures include the glucagon receptor (GCGR) [15, 16] and the corticotropin releasing factor 1 receptor [14]. It has been proposed that the GCGR ECD stays close to its TMD in the apo-state and that the binding of peptide agonist rearranges the ECD–TMD assembly [24]. Residues surrounding the extracellular pocket of the TMD were shown to be critical for glucagon binding and activation of GCGR, leading to a proposed model that the N-terminus of the peptide hormone may insert deeply into the extracellular pocket of the TMD for receptor activation [15]. In this model, the ECD is proposed to adopt a ‘stand up’ position, in which the ECD is almost perpendicular to the membrane surface when a hormone peptide binds to the receptor [15, 24]. This ‘stand-up’ activation model fits well with the general framework of the ‘two domain’ mechanism for ligand binding and receptor activation of class B GPCRs, where the ECD serves as an affinity trap to interact with the C-terminal portion of peptide hormones, allowing the N-terminus of peptide hormones to insert into the TMD to activate the receptor [19, 20, 23–26].

Although the ‘two domain’ mechanism is a generally accepted model for hormone binding and activation for class B GPCRs, different requirements of the ECD for the receptor activation exist among members of this family of GPCRs [27]. The ECDs of corticotrophin-releasing factor receptor 1, parathyroid hormone receptor and pituitary adenylate cyclase activating polypeptide type 1 receptor appear to solely serve as affinity traps for peptide ligands and their functions can be bypassed by covalently linking peptide ligands to the receptor TMD. In contrast, the ECD is required for activation of GCGR and GLP-1R, even when the peptide hormone is covalently linked to the receptor, suggesting that the ECD plays different roles in activation of different members of class B GPCRs [27]. The ECD requirement for the activation of GCGR and GLP-1R suggests that the ECD plays a direct role in activation of these receptors in addition to peptide binding.

In this paper, we report the mechanistic basis for the ECD requirement for activation of GLP-1R through comprehensive peptide deletion and receptor mutagenesis studies. Unexpectedly, we found that the first five amino acid residues at the N-terminus of GLP-1 and EX4 are dispensable for wild-type (WT) GLP-1R activation in both cyclic adenosine monophosphate (cAMP)/G protein signaling and arrestin-mediated pathways when they are fused to the receptor. This data clearly challenges the model of insertion of the N-terminus of peptide hormones into the TMD for receptor activation. Furthermore, we demonstrate that GLP-1R C347K and C347R mutant proteins can be activated by a non-specific five residue linker in the complete absence of GLP-1 or EX4 sequences, yet this activation remains completely dependent on the ECD, suggesting that the ECD plays a much more dominant and direct role in the receptor activation. In addition, we show that a membrane-attached GLP-1R ECD can mediate hormone activation of the GLP-1R TMD in trans, suggesting a direct GLP-1R ECD-TMD interaction as a mechanistic requirement for receptor activation. Finally, we found that the C347K/R mutant receptor is unexpectedly Gs-biased and defective in arrestin-biased signaling. C347 is located in the intracellular loop 3, which is a key binding site for G protein interaction and this unexpected discovery provides a basis for understanding the GPCR biased signaling.

## Results

### Both G protein and arrestin pathways of GLP-1R can be activated by N-terminally truncated GLP-1 or EX4 that are fused to the receptor

Peptide hormones are agonists of class B GPCRs that induce the receptors to recruit either stimulatory G proteins (Gs), leading to elevation in intracellular cAMP levels, or G protein-coupled receptor kinases for receptor phosphorylation, leading to arrestin recruitment and arrestin-mediated pathways. Previous literature reported that the N-terminus of a class B peptide hormone functions as an agonist to mediate hormone-induced receptor activation [28, 29]. In agreement, a truncated EX4 lacking its two most N-terminal residues [Ex4(3–39)] was reported as a high-affinity receptor antagonist [30]. In our studies, we determined the GLP-1R signaling activity of both the N-terminally truncated peptide hormones GLP-1 and EX4 when fused to the N-terminus of the receptor to overcome the selective recognition and low affinity of the small peptide by the receptor. The hormone-tethered receptors were expressed as fusion proteins with N-terminal IgG signal peptides to ensure proper membrane localization.

**Figure 1:**
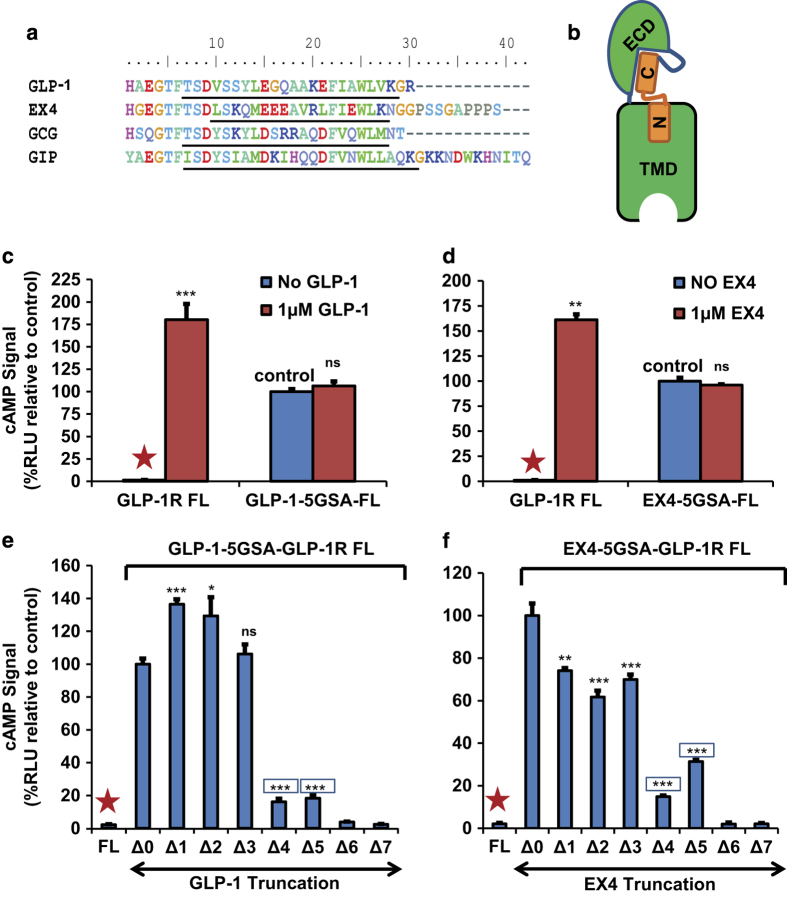
GLP-1R fused with truncated EX4 or GLP-1 peptide can activate G protein signaling pathway. (**a**) Amino acid sequence alignment of GLP-1 family peptides: glucagon-like peptide-1, exendin 4, glucagon and gastric inhibitory polypeptide (GIP). Underlined are the α-helical regions of the peptides. (**b**) Cartoon representation of the hormone-fused receptor construct. The hormone (orange) is shown as two bars representing the N- and the C-portions of the peptide, while the dark blue line represents the linker between the ECD and hormone or between ECD and the TMD. (**c**, **d**) The G protein or cAMP signals produced by full-length receptor (FL), or the full-length receptor fused with full-length EX4 or GLP-1, in the absence or presence of exogenously added GLP-1 or EX4. (**e**, **f**) The G protein or cAMP signals induced by the receptor fused with full length or sequentially truncated EX4 or GLP-1. Activities are represented relative to those of the full-length constructs GLP-1-5GSA-FL or EX4-5GSA-FL, which were set to 100%. The ‘Δ number’ indicates the number of residues deleted from the N terminus of hormone peptide. Error bars=s.d. All experiments were performed as three independent transfection experiments; two-tailed Student’s *t* test was used to determine non-boxed *P*-values for data point versus control (Δ0) or boxed *P*-values for data point versus negative control (FL, marked by red asterisks): NS>0.05,*⩽0.05; **⩽0.01; ***⩽0.001; ****⩽0.0001.

**Figure 2:**
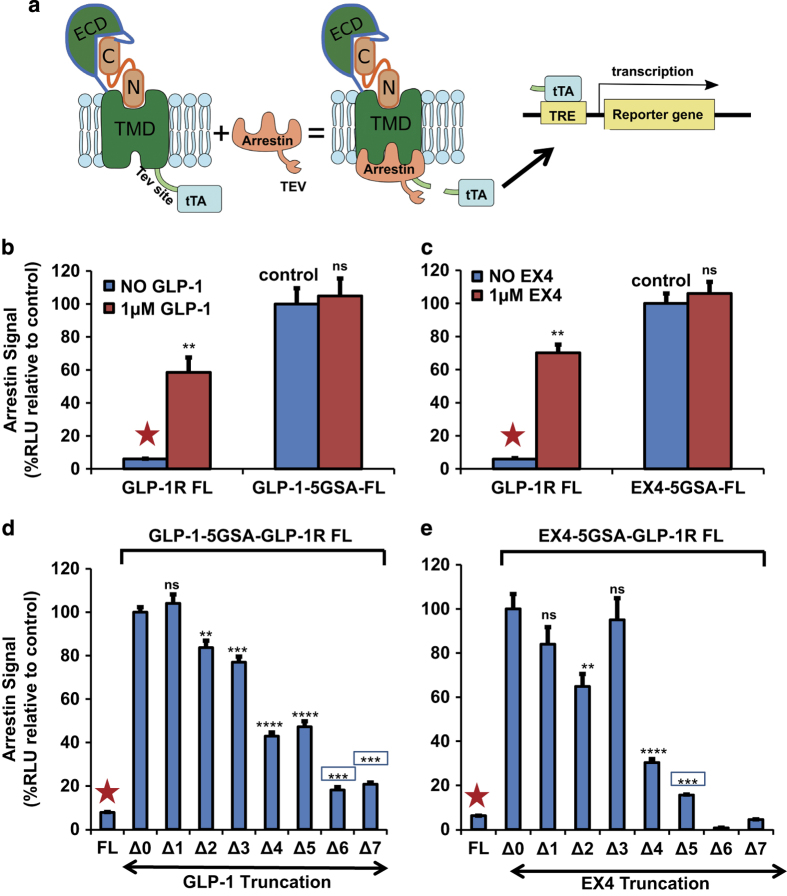
GLP-1R fused with truncated EX4 or GLP-1 peptide can activate the arrestin signaling pathway. (**a**) The design of the ‘tango assay’ to detect arrestin binding through luciferase reporter signals. The tTA/TRE-luc reporter signal was used to compare the binding capacity of the different constructs to β-arrestin 1. TEV site, tobacco etch virus protease cleavage site; tTA, transcriptional activator. (**b** and **c**) The arrestin binding activity of full-length GLP-1R or GLP-1R fused with full-length EX4 or GLP-1 in the absence or presence of hormones. (**d** and **e**) Arrestin binding signals induced by sequentially N-terminally truncated EX4 or GLP-1 peptides fused to the receptor. All signals were calculated relative to the control signal (full-length GLP-1-5GSA-FL or EX4-5GSA-FL, Δ0), which was set to 100%. The ‘Δ number’ indicates the number of N-terminally truncated residues of the hormone peptides. Error bars=s.d. All experiments were performed as three independent transfection experiments; two-tailed Student’s *t* test was used to determine non-boxed *P*-values for data point versus control (Δ0) or boxed *P*-values for data point versus negative control (FL, marked by red asterisks): NS>0.05,*⩽0.05; **⩽0.01; ***⩽0.001; ****⩽0.0001.

We utilized this peptide fusion method to evaluate agonist potencies of GLP-1 family peptides (Figure 1a) for GLP-1R activation by determining cAMP levels (Gs activity) and β-arrestin recruitment signaling [31] in cell-based reporter assays (Figures 1 and 2). β-arrestin recruitment by GLP-1R is detected by a tango assay [32], in which the receptor is fused with a tobacco etch virus (TEV) protease cleavage site and the transcriptional activator tTA, while arrestin is fused with TEV protease. Ligand binding to GLP-1R induces recruitment of the arrestin-TEV fusion protein to the receptor, leading to cleavage at the TEV site and the release of tTA to activate the reporter (Figure 2a). To examine the requirement of the peptide hormone N-terminus for receptor activation, we fused full length or N-terminally truncated GLP-1 or EX4 peptide via a 15 amino acid linker (five copies of Gly-Ser-Ala [5xGSA]) to the N-terminus of GLP-1R (Figure 1b). The receptors fused with full-length GLP-1 or EX4 were constitutively active in both G protein/cAMP pathway (Figure 1c and d) and β-arrestin pathway (Figure 2b and c). Addition of exogenous EX4 or GLP-1 peptides even at high concentration (1 μm) did not further increase G protein/cAMP and arrestin recruitment signaling levels, suggesting that the fusion receptors were fully occupied by the fused peptides. When compared with unfused receptors activated by saturated concentration (1 μm) of GLP-1 or EX4, the fused receptors had slightly lower G-protein/cAMP signaling levels (~60% of fully activated unfused receptor, Figure 1c and d), but exhibited higher arrestin recruiting signaling levels (~150% of the fully activated unfused receptor, Figure 2b and c).

To determine the role of the N terminus of EX4 or GLP-1 peptide hormone in GLP-1R activation, we sequentially deleted amino acids from the N terminus of the fused peptide. When the first amino acid histidine was deleted from the fused GLP-1(7-36) or EX4(1–39), the cAMP signaling of GLP-1 increased to 136%, and that of EX4 decreased to 74% relative to the full-length fusion receptors (Figure 1e and f). The same truncated GLP-1 or EX4 fusion receptors showed little effects on arrestin activity relative to the full-length fusion receptors (Figure 2d and e). Those findings suggest that the first histidine residue of GLP-1 and EX4 only plays a marginal role in activation of the GLP-1R when the peptide is fused to the receptor. Deletion of an additional two residues from GLP-1 or EX4 retains most activity of the fusion receptors in cAMP and arrestin signaling levels, indicating that the removal of the first three amino acids had little effect on the function of GLP-1 and EX4 when they are fused to the receptor. Truncation of the fourth and fifth residues of GLP-1 reduced the cAMP activity to 16–18%, but retained nearly 50% of the arrestin signal. Removal of the first six and seven residues of GLP-1 and EX4 nearly abolished the cAMP signal (Figure 1e and f); however, the GLP-1 peptide still maintained a decent 18–20% activity in arrestin recruitment (Figure 2d and e). All EX4 constructs above have almost the same expression level no matter whether based on the glycosylated or the non-glycosylated receptor relative to the level of β-actin used for normalization (Supplementary Figure S1A).

**Figure 3:**
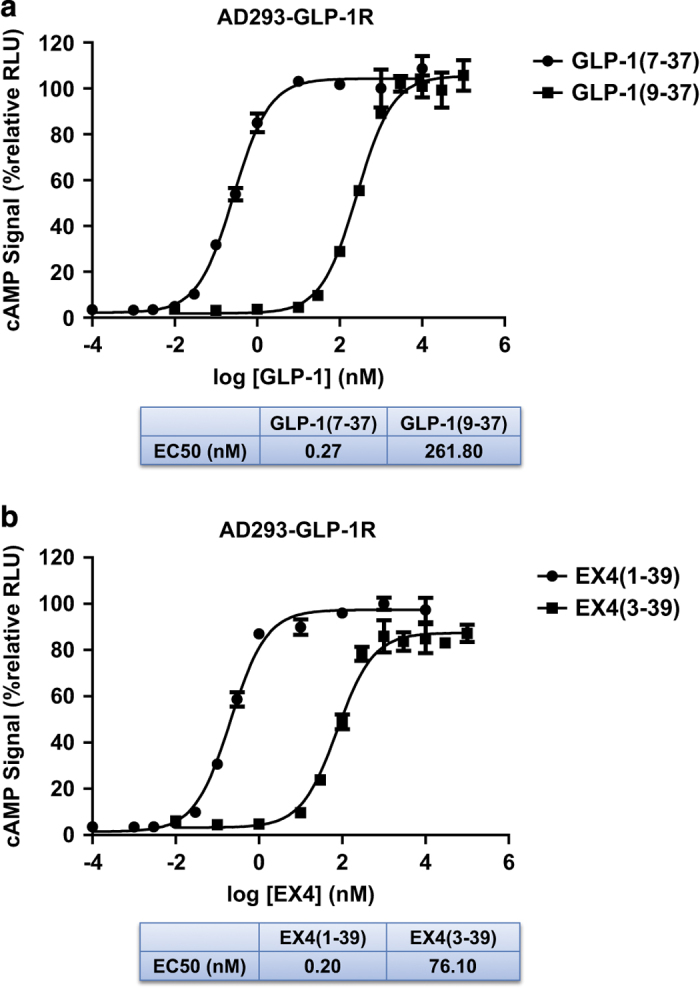
Receptor EX4/GLP-1 dose-cAMP response curves. Dose-dependent cAMP signals were produced by activation of the full-length GLP-1R receptor in AD293 cells that stably express human GLP-1R and CRE-NanoLuc luciferase (pNL-NlucP-CRE-Hygro) reporter (see the ‘Materials and methods’ section). The activity of the receptor exposed to 1 μm EX4 or GLP-1 was set to 100%. (**a**) cAMP signals in response to full length (residues 7–37) and truncated (residues 9–37) GLP-1. (**b**) cAMP signals in response to full length (residues 1–39) and truncated (residues 3–39) EX4. All values are means±s.e.m. of two independent experiments, each conducted in triplicate.

The above deletion data in our fusion system is inconsistent with the current model of GLP-1R activation, in which insertion of the N-terminus of the peptide hormone into the receptor TMD is required for receptor activation. To test whether the truncated peptides are only functional in the context of the fusion receptors, we used synthetic peptides of GLP-1 and EX4 that lack the first two residues, GLP-1(9-39) and EX4(3–39). Both of these peptides can activate the receptor to the same maximal level as their corresponding full-length peptides (Figure 3a and b). The only difference is in their potencies to activate the receptor, with the potency (EC_50_ value) of the full-length EX4 peptide being ~380-fold higher than that of the truncated peptide and that of the GLP-1 peptide being ~1000-fold higher than its truncated version. These data demonstrate that the non-fused EX4 or GLP-1 peptide with deletion of the first two amino acids still retain the full capacity to promote the cAMP signal when these peptides are present in higher concentrations. These non-fused peptide data are also inconsistent with the model of receptor activation by the insertion of the peptide N-terminus into the GLP-1R TMD, but they are consistent with the data obtained with the peptide-fused receptors.

### Mutation of cysteine 347 of GLP-1R to lysine or arginine transforms the receptor into a G protein-biased receptor and rescues the loss-of-function of the truncated peptides

**Figure 4:**
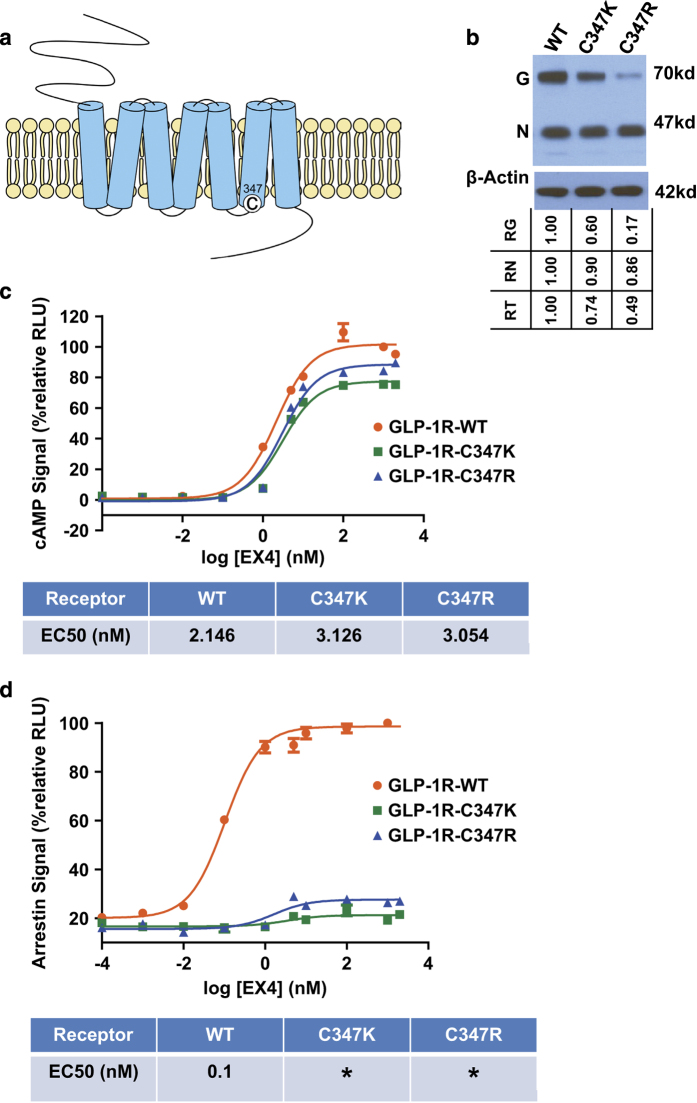
C347K/R mutation transforms full-length GLP-1R into a G-protein biased receptor. (**a**) Position of C347 in GLP-1R. (**b**) Expression levels of WT and C347K/R GLP-1R detected by immunoblotting using anti-FLAG antibody for detection and β-actin antibody for normalization. G: glycosylated receptor, N: non-glycosylated receptor. ‘RG’ refers to normlalized glycosylated receptor signal relative to WT (WT set as 1.00); ‘RN’ refers to normlalized non-glycosylated receptor signal relative to WT. ‘RT’ refers to normlalized total receptor signal relative to WT. Expression of β-actin is used for normalization (see the ‘Materials and methods’ section). (**c** and **d**) EX4(1–39) dose-response curve for cAMP (**c**) or arrestin binding signal (**d**) in WT or C347K or R mutated GLP-1R. C347K/R mutant receptors do not activate arrestin signal. All values are means±s.e.m. of two independent experiments, conducted in triplicate. The activity of the receptor exposed to 1 μm EX4 was set to 100%. *: EC50 unavailable.

**Figure 5:**
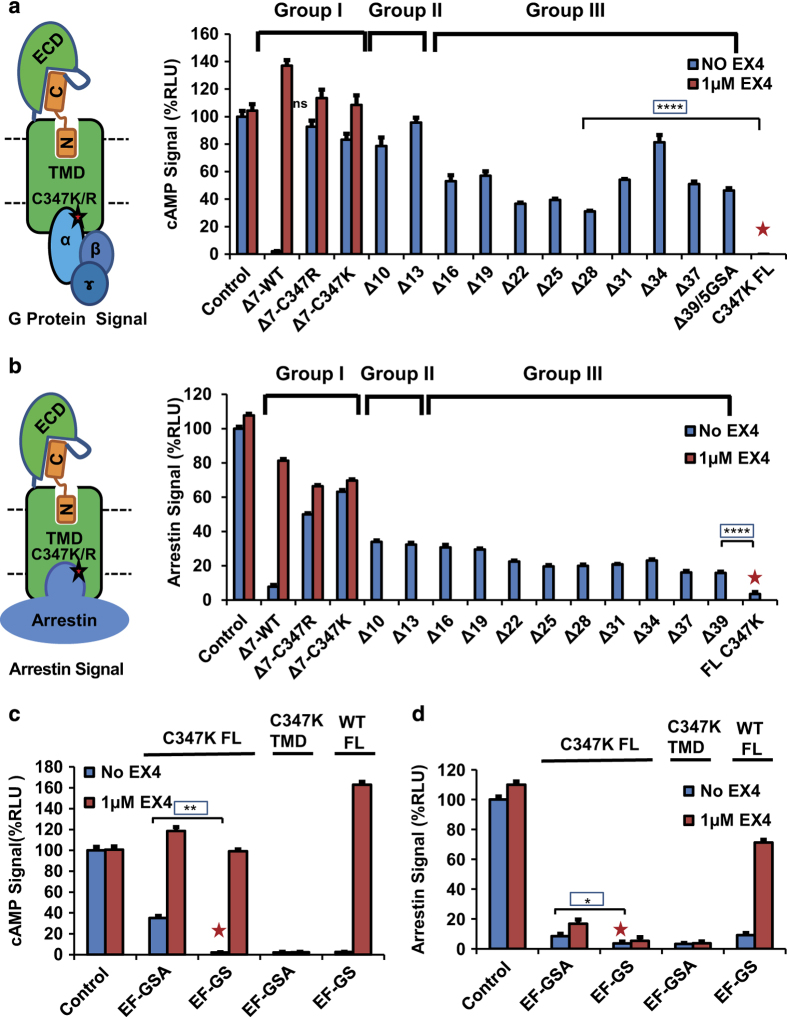
GLP-1R C347K can be activated by a non-specific five residue linker that is devoid of EX4 or GLP-1 sequence. (**a**) cAMP signal induced by truncated peptide fused to full-length GLP-1R with C347K mutation. Left, a cartoon presentation of G protein activation by the EX4-5GSA-GLP-1R C347K/R fusion receptor. Group I: EX4(8-39)-5xGSA-GLP-1R WT, and C347K/R mutant. Group II: EX4-5xGSA-GLP-1R C347K with truncations of 10 or 13 N-terminal EX4 residues. Group III: EX4-5xGSA-GLP-1R C347K with truncations of 16–39 (39=all) EX4 residues. (**b**) Arrestin binding signals induced by sequentially truncated peptide hormone fused to full-length GLP-1R C347K. Left, a cartoon presentation of arrestin recruitment by the same EX4-5xGSA-GLP-1R C347K/R fusion system. Groups I, II and III represent the arrestin binding signals induced by the hormone-GLP-1R mutant systems described in **a**. (**c** and **d**) The cAMP (**c**) or arrestin binding (**d**) signals induced by EFGSA linker fused to the N terminus of WT or C347K full-length GLP-1R or GLP-1R TMD. ‘Δ number’ indicates the number of N-terminal residues that were deleted from EX4. Non-fused or EF-GS fused full-length GLP1R C347K was used as negative control (marked by red asterisks). Error bars=s.d., all experiments were performed as three independent transfection experiments. Two-tailed Student’s *t* test was used to determine *P*-values, which show no significant difference (NS>0.05) in activation between WT receptor fused with full length EX4 (control) and ∆7-C347K, but there is significant difference in activity between C347K fusion with truncation peptide (group III) and non-fused C347K full-length receptor (marked by red asterisks), which is not active in the absence of exogenous peptide (boxed *P-*values: *⩽0.05; **⩽0.01; ***⩽0.001; ****⩽0.0001).

GPCRs have been viewed as ‘allosteric machines’ [33] because many allosteric modulators have been identified binding outside the orthosteric pocket of the physiological activators to modulate endogenous agonist binding and function. 4-(3-(Benzyloxy)phenyl)-2-ethylsulfinyl-6 (trifluoromethyl)pyrimidine (BETP) is such a small molecule that covalently attaches to C347 in the intracellular loop 3 near the beginning of the transmembrane helix 6 (TM6; Figure 4a) and functions as a positive allosteric modulator for GLP-1R [34, 35]. Although mutating C347 to positively charged lysine or arginine decreased the expression levels of the mutated receptors (Figure 4b), the ability of these mutated receptors to activate G-protein/cAMP signaling by GLP-1 was largely unaffected (Figure 4c). However, the ability of these mutated receptors to activate arrestin signaling was nearly abolished (Figure 4d), suggesting that these mutated receptors are G protein-biased receptors. For the WT receptor, deletion of the first seven N-terminal residues of the fused peptide largely abolished the agonist activity of the peptide hormones in both signaling pathways (Figures 1e, f and 2d, e). Surprisingly, introduction of the C347K/R mutation in the receptor rescued the activity of the seven residue N-terminal truncation in the EX4 peptide (Figure 5a and b group I, expression level shown in Supplementary Figure S1B). Like the WT receptor, the non-fused full-length C347K receptor remained inactive in the absence of exogenous peptides (Figure 5a, C347K FL lane labeled with red asterisk, see also Figure 4c and d). These data suggest that for the C347K mutated receptor, the first seven residues of EX4 are not required for GLP-1R activation when EX4 is fused to the receptor. It is worth to note that the C347K mutated receptor is G-protein biased for the non-fused EX4 ligand but is unbiased when EX4 is fused to the receptor (Figures 4c, d and 5a, b), suggesting that biased and unbiased signaling are very sensitive to receptor context and to the fused/unfused status of EX4 peptide.

To further determine the minimum length of the peptide hormones whose activity can be rescued by C347K mutation, we performed cell-based assays using C347K mutated GLP-1R fused with sequentially truncated EX4 peptides. We were surprised that the deletion of 7–13 N-terminal residues retained almost all G protein activity when compared with the WT receptor fused with full-length EX4 (Figure 5a, group II versus Δ0-WT (control)). Furthermore, 30–80% of G protein signaling activity was retained by peptides with truncations of 16–39 residues compared with the full-length EX4 fused receptor (group III versus Δ0-WT (control)). The ∆39 receptor completely lacked all EX4 residues, but contained residues of the 5GSA linker (Figure 5a, group III, expression levels shown in Supplementary Figure S1C). This is contradicting the notion that the N terminus of the peptide is critical for receptor activity. We further removed the GSA linker residues and found that a single GSA with two additional EF residues from the *Eco*RI cloning site is sufficient to activate the C347K mutant receptor at 30% of the activity of full-length peptide fusion receptor (Figure 5c, expression levels shown in Supplementary Figure S2A). Deletion of one additional residue (EF-GS construct) abolished the activation of the receptor, yet the receptor can be activated by exogenous EX4 to a comparable level, indicating the receptors were functionally expressed (the expression levels also shown in Supplementary Figure S2A). The intrinsic activation of the EF-GSA fused receptor still required the ECD as the fusion receptor lacking the ECD (‘C347K TMD’) was not activated (Figure 5c) even though it was well expressed (Supplementary Figure S2A).

Interestingly, the arrestin activity of the mutant GLP-1R showed a similar profile, but at lower levels, as those of the G protein activity when the same truncated peptides were used (Figure 5b). The C347K mutation did not rescue as much of the lost activity in the arrestin pathway as it did in the G protein pathway (Figure 5b, groups II and III). The same non-specific peptide EF-GSA fused to the N-terminus of the receptor failed to strongly increase arrestin signaling compared with the non-specific peptide EF-GS, even when free EX4 was added (Figure 5d), providing further support that the C347K mutation changes GLP-1R to a G protein-biased receptor that favors G protein-associated signaling while it hinders the arrestin signaling.

Together, the above data demonstrate that the non-specific five residue linker EF-GSA fused to the C347K mutated receptor is sufficient to activate the receptor and, importantly, this activation requires the ECD. The activation of C347K GLP-1R by a peptide that is completely devoid of the sequence of GLP-1 and EX4, but still requires the ECD, suggests that the ECD plays a direct role as an ‘intrinsic’ agonist to activate this mutated receptor.

### The ECD can activate GLP-1R in trans in the presence of intact peptide hormone

**Figure 6:**
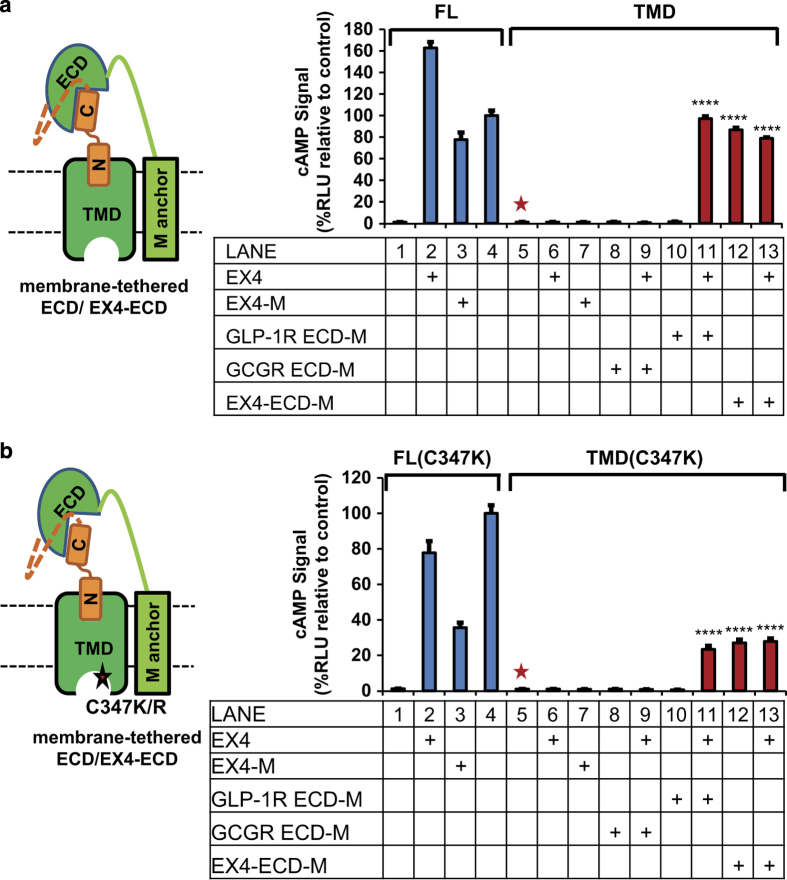
The ECD is a positive regulator and can activate GLP-1R in trans. (**a**, **b**) The ECD activates GLP-1R (**a**) or GLP-1R C347K (**b**) in trans. Left, cartoon presentation of the TMD (WT/C347K) co-expressed with membrane-tethered ECD (ECD-M) or EX4-ECD fusion (EX4-ECD-M). Dashed orange line indicates the 5xGSA linker between ECD and full-length EX4 peptide. Right, the cAMP signals produced by the TMD in the presence of GLP1R ECD (red bars) versus those induced by the intact GLP-1R or GLP-1R TMD co-expressed with membrane-tethered GCGR ECD (blue bars). All signals are indicated as relative to control (EX4-5xGSA-GLP-1R FL WT, lane 4), which was set as 100%. M: membrane tethered. Error bars=s.d., all experiments were performed as three independent transfection experiments. Two-tailed Student’s *t *test was used to determine *P*-values for data points versus control (TMD without co-expressed ECD construct, lane 5, marked by red asterisk). ****⩽0.0001.

We next wanted to determine the mechanism by which the ECD can serve as a positive regulator to activate the receptor. It was previously reported that membrane-tethered class B peptide hormones can trigger dose-dependent activation of G protein signaling of their cognate co-expressed GPCRs [36]. We modified this approach to further investigate the roles of the ECD in GLP-1R activation. As shown in Figure 6a, we tethered the ECD to a membrane anchor either with or without an N-terminally fused EX4 (ECD-M and EX4-ECD-M, respectively). For the full-length GLP-1R, exogenous EX4 or membrane-tethered EX4 induced strong G-protein activation signals (Figure 6a, lanes 1–3). The receptor TMD alone was not activated by exogenous EX4 or by co-expressed EX4-M (Figure 6a, lanes 5–7). Importantly, when the GLP-1R TMD was co-expressed with the GLP-1R ECD, the TMD can be activated by exogenous EX4 (Figure 6a, lanes 10–11). This activation is specific for the GLP-1R ECD as the GLP-1R TMD is not activated by EX4 when co-expressed with the GCGR ECD (Figure 6a, lanes 8–9). In addition, the GLP-1R TMD can be activated by the membrane-tethered EX4-ECD independent of the presence of exogenous EX4 (Figure 6a, lanes 12–13), but cannot be activated by the membrane-tethered EX4 without the GLP-1R ECD (Figure 6a, lane 7). Parallel data were obtained with the C347K receptor (Figure 6b). Together, these data further support that the ECD is required for GLP-1R activation and can be provided in trans as a membrane-tethered fusion protein.

**Figure 7:**
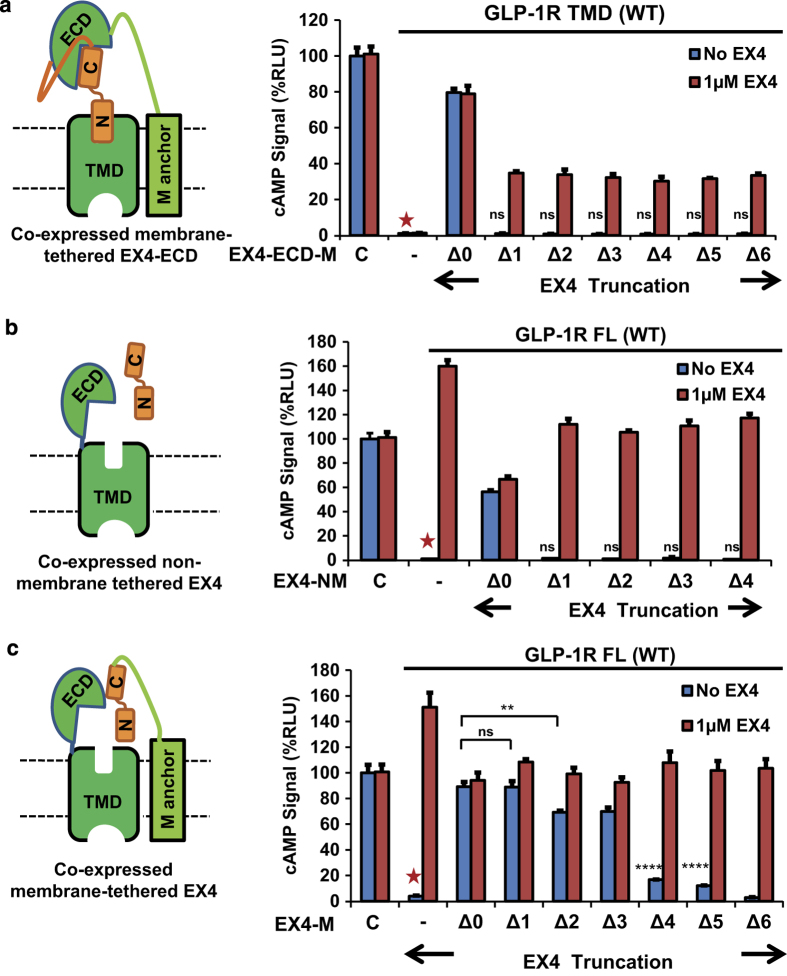
Trans activation requires the N-terminus of EX4. The cAMP signal stimulated by (**a**) GLP-1R TMD co-expressed with full length or truncated EX4-ECD-M; (**b**) full-length GLP-1R co-expressed with non-membrane tethered full length or truncated EX4 peptides and (**c**) full-length GLP-1R co-expressed with membrane-tethered full length or truncated EX4. All signals are indicated as relative to control (EX4-5xGSA-GLP-1R FL WT, labeled C), which was set to 100%. Left, cartoon representations of constructs. ‘−’, absence of EX4 construct. ‘NM’, non-membrane tethered. ‘M’, membrane tethered. Error bars=s.d., all experiments were performed as three independent transfection experiments; two-tailed Student’s *t *test was used to determine *P*-values for data points versus GLP-1R construct lacking EX4 (marked by red asterisk): NS>0.05; *⩽0.05; **⩽0.01; ***⩽0.001; ****⩽0.0001.

We further tested whether a truncated peptide hormone fused to the N terminus of the ECD can activate the receptor in trans. To our surprise, the deletion of only the first residue abolished the agonist activity of EX4-ECD (Figure 7a). Similarly, the truncated peptides when co-expressed in trans and not receptor- or membrane-tethered were not functional in the activation of the full-length receptor (Figure 7b). This is in contrast to the fully retained activity of the Δ3 peptide hormone when provided in cis as fusion with the full-length receptor (Figures 1 and 2). The inability of these N-terminal EX4 deletions to activate GLP-1R probably resulted from low co-expression level of the peptide hormone (EX4-NM) because the full-length GLP-1R can be activated by high concentrations of exogenously added EX4 or GLP-1 with deletion of two N-terminal residues (Figure 3). Furthermore, when EX4 was co-expressed as a membrane-tethered protein, deletion of its first three residues had little effect on activation of the full-length GLP-1R, suggesting that the membrane-tethered expression could effectively increase the local concentrations of the fusion proteins (Figure 7c), which further supports that the N-terminal residues of EX4 are not required for activation of GLP-1R.

### The extracellular loops of the GLP-1R serve as the interface of the TMD, the ECD and the peptide hormone

**Figure 8:**
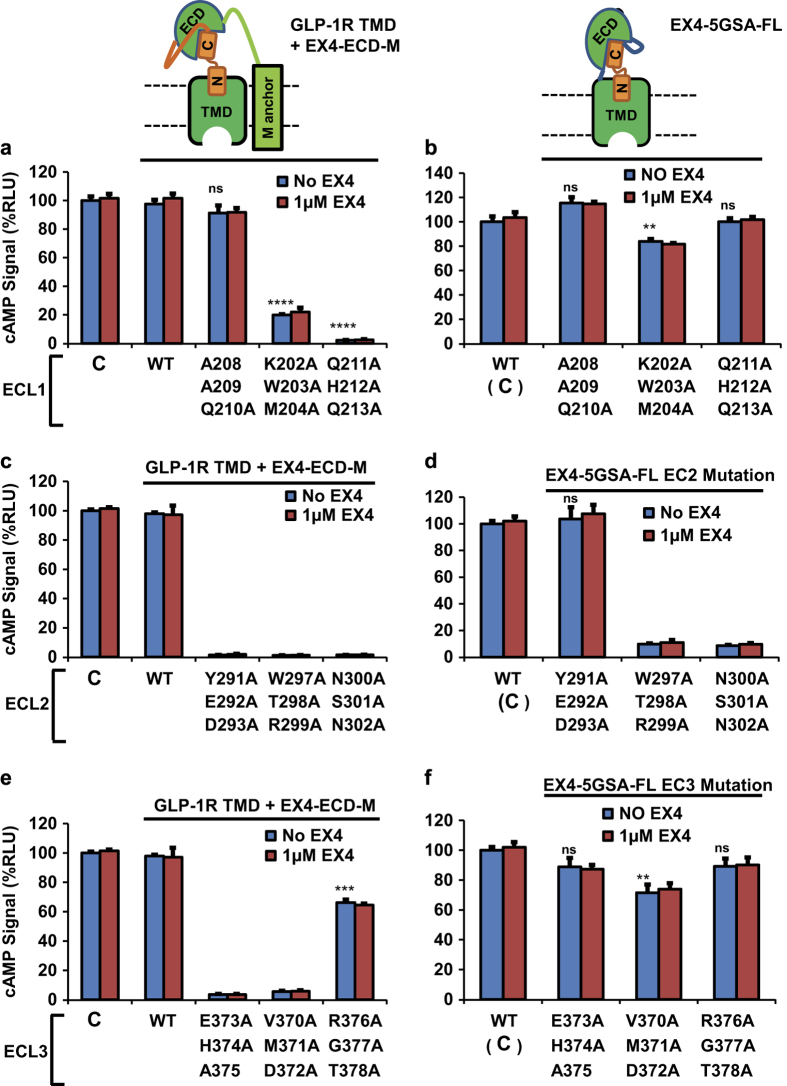
The interaction interface between ECD and TMD localizes to the ECL of GLP-1R. Top left: cartoon representation of receptor TMD co-expressed with membrane-tethered EX4-ECD fusion (EX4-ECD-M); this construct has been used in **a**, **c** and **e**. Top right: cartoon representation of the hormone-fused receptor; this construct has been used in **b**,** d** and **f**. (**a**, **c** and **e**) cAMP signal produced by EX4-ECD-M and GLP-1R TMD with indicated mutations in ECL1, 2 and 3. (**b**, **d** and **f**) cAMP signal produced by EX4-5xGSA-GLP-1R FL construct harboring mutations in the same sites as the constructs in (**a**, **c** and **e**). All signal are indicated as relative to control (EX4-5xGSA-GLP-1R FL WT, labeled as C in **a**, **c** and **e**, as C with bracket in **b**, **d** and **f**), which was set to 100%. EX4-ECD-M: membrane-tethered EX4-ECD fusion. Error bars=s.d., all experiments were performed as three independent transfection experiments; two-tailed Student’s *t *test was used to determine *P*-values for data points versus WT: NS>0.05; *⩽0.05; **⩽0.01; ***⩽0.001; ****⩽0.0001. See Supplementary Figure S3B–D for activities normalized to expression levels.

The ability of the ECD to activate the GLP-1R TMD in trans in the presence of EX4 suggests a physical interaction between the ECD, TMD, and the peptide. We set out to identify the key interface residues that facilitate these interactions by mutagenesis. Previously published site-directed mutagenesis of GLP-1R [37–40] suggested that the extracellular loops (ECL) of the receptor may be involved in ECD-TMD interaction. We therefore designed an alanine scanning mutagenesis, in which we mutated blocks of three consecutive residues in ECL 1, 2 or 3, to probe the roles of the ECL residues in the interaction between ECD and TMD. Figure 8 shows the G protein signaling activity of mutated GLP-1R TMD receptors co-expressed with membrane tethered EX4-ECD fusion protein (Figure 8a, c and e) and mutated full-length receptors with N-terminally fused EX4 (Figure 8b, d and f).

The ECL1 and ECL3 mutations did not cause a dramatic reduction of G protein activation when EX4 was linked to the full-length receptor (Figure 8b and f). In contrast, the mutations significantly reduced the receptor activity when EX4-fused ECD was expressed separately from the TMD, especially for the Q211A/H212A/Q213A, E373A/H374A/A375 and V370A/M371A/D372A mutations, which nearly abolished the receptor activity (Figure 8a and e, expression levels shown in Supplementary Figure S2B and D and the normalized activity shown in Supplementary Figure S3A and C). The ECL2 mutations W297A/T2-98A/R299A and N300A/S301A/N302A, which have the same expression levels as WT (Supplementary Figure S2C), however, largely reduced the G protein activation of both the full-length receptor and the TMD co-expressed with EX4-fused ECD (Figure 8c and d; the activity normalized to expression levels is shown in Supplementary Figure S3B). In contrast, the ECL2 mutation Y291A/E292A/D293A, which had little effect in the context the full-length receptor fused with full-length peptide, caused a total loss of function in the context of the TMD co-expressed with the EX4-fused ECD. Together, these data demonstrate that mutations in ECLs can specifically affect the activation of GLP-1R TMD by EX4-ECD when provided in trans, suggesting that these mutated residues could serve as the contact sites between ECD and TMD.

## Discussion

**Figure 9:**
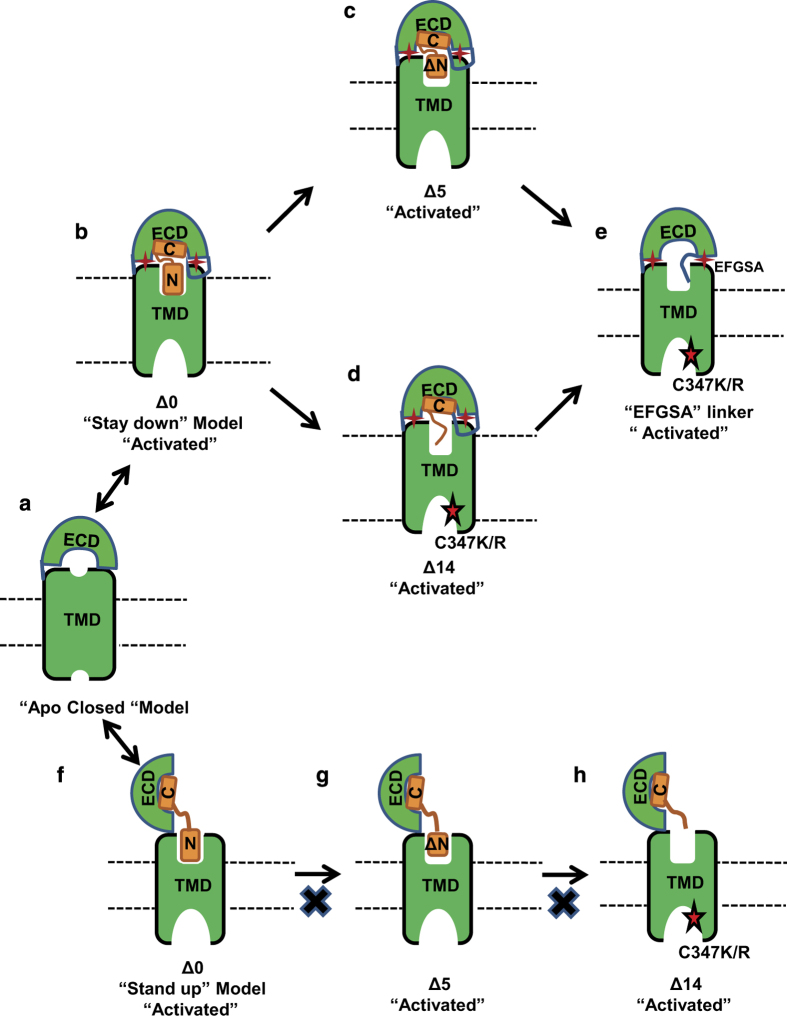
A cartoon presentation of activation models of GLP-1R. (**a**) The closed-state apo receptor, which is auto-inhibited by its ECD-TMD interactions. (**b–e**) An ECD intrinsic agonist model for activation of GLP-1R, which accounts for the activation of the TMD by the ECD in trans, and for activation of GLP-1R by N-terminal deletion peptides and GLP-1R C347K/R by a linker peptide devoid of EX4 sequence. In this intrinsic agonist model, the receptor is activated by an allosteric interaction of the ECD with the TMD. (**f–h**) The classic stand-up activation model of GLP-1R, which cannot account for activation of the receptor by N-terminal deletion peptides. Solid red stars represent an interaction between ECD and TMD, the red stars with black outline indicate activation by the C347K/R mutation.

In this paper, we present the surprising result that the N-terminal residues of GLP-1 and EX4 are not required for activation of GLP-1R when the peptides are fused to the receptor. Furthermore, for the C347K mutated receptor, the GLP-1 and EX4 sequences are completely dispensable for receptor activation and a non-specific five residue linker is sufficient to activate the full-length receptor, but not the TMD of the receptor. Consistent with our previous observations, the GLP-1R ECD is required for activation and the current data show that the ECD can be provided as a trans fusion protein. Based on these findings, we propose an ‘intrinsic agonist’ model of the ECD for GLP-1R activation, in which the ECD is the major determining factor in activation of GLP-1R (Figure 9). It has been proposed that the GCGR ECD stays in close contact with the TMD in the apo state to inhibit the receptor activation [24] (model in Figure 9a). One of the most important features of the proposed ‘intrinsic agonist’ model of the ECD is that the ECD remains in close contact with the receptor TMD, marked as ‘stay down’ in Figure 9b, even after ligand binding. Ligand binding switches the receptor from the auto-inhibited state to the auto-activated state by releasing the intrinsic agonist activity of the ECD. Thus, in this model, the peptide serves as an allosteric modulator of the ECD conformation and ECD-TMD interaction, which then activates the receptor.

The ‘intrinsic agonist’ model of the ECD for the receptor activation is consistent with the ability of the N-terminally deleted hormone peptides to activate GLP-1R (Figure 9c and d) and the complete independence of GLP-1 and EX4 sequence for activation of the C347K/R mutated receptor (Figure 9e). In the case of GCGR, a stand-up model has been proposed for ligand binding, in which the receptor switches from the apo-closed conformation of the ECD to an open conformation [15, 24], in which the ECD is almost perpendicular to the membrane surface (model in Figure 9f). In this conformation, the C-terminal helix of the hormone binds the ECD to allow the N-terminal portion of the peptide to insert into the TMD, an event that is proposed to be critical for activation of the receptor (Figure 9f). However, this activation model by the N-terminal insertion of peptides is incompatible with our deletion data, which show that up to five N-terminal residues of GLP-1 and EX4 are dispensable for activation of GLP-1R when these peptides are fused to the receptor (Figure 9g and h). The activation of GLP-1R by N-terminally deleted GLP-1 and EX4 is not restricted to the fusion peptides but also occurred with non-fused, exogenously added peptides. Both GLP-1 and EX4 with deletion of two N-terminal residues can achieve almost the full activation of GLP-1R when they are present at 350- or 1000-fold higher concentrations, respectively. This data is consistent with the N-terminal residues of GLP-1 and EX4 not being required for the receptor activation when the peptide is fused to the receptor.

The ‘intrinsic agonist’ model of the ECD implies that the ECD remains in close interaction with the TMD during ligand binding and receptor activation. This is supported by the fact that the ECD can be provided separately as a trans-fusion protein for receptor activation (Figures 6 and 7). Mutations in specific residues in the ECL1, 2 and 3 can abolish the trans-activation of the ECD, suggesting that these residues could be the potential binding sites for the ECD-TMD interactions. It is well established that in the apo-state the ECD also forms a close interaction with the TMD and this interaction inhibits receptor activation, because mutations in the ECL3 residues of the calcitonin gene-related peptide receptor and GCGR have resulted in significant increases in both basal and ligand-induced activity [41–44]. The close ECD-TMD interaction in the apo-state is reminiscent of the state in the recent structure of the smoothened receptor, which shows extensive interactions of its ECD with the ECLs of its TMD [45]. However, the ECD–TMD interactions in the apo-state must be different from those in the ligand-bound state. In the apo-state, the ECD serves as a negative regulator whose interaction with the TMD inhibits the basal activation of the receptor [42, 46]. In contrast, in the presence of ligand, the ECD is a positive activator whose interaction with the TMD results in a different conformation that leads to receptor activation. In this case, the peptide hormone serves as a positive allosteric modulator whose binding to the receptor switches the ECD-TMD conformation from an auto-inhibitory state to an active state.

In addition, it is a surprise that the C347K/R mutation converts GLP-1R into a Gs-biased receptor that is defective in arrestin-biased signaling (Figure 4). GPCR mutants are more prone to be beta-arrestin biased rather than Gs-protein biased as G-protein binding requires a much more pronounced outward movement in TM6 than arrestin binding [47, 48]. C347 is located in the intracellular loop 3 near the cytoplasmic side of TM6, which is a key binding site for G protein interaction. It is possible that this mutation unlocks the inactive conformation state of TM6 and allows the outward movement of TM6 to accommodate the Gs protein, but not arrestin. This explanation is consistent with the fact that the C347K/R mutated GLP-1R can be activated by a non-specific 5-residue linker, whose sequence is totally unrelated to GLP-1 and EX4. Thus, the C347K/R mutation lowers the energy barrier of GLP-1R to be activated by its ECD and this lower energy barrier can be overcome by a weak interaction of the non-specific five residue linker, which in this case serves as a positive allosteric modulator for the mutated receptor.

A recent study [49] demonstrates that the extracellular surface is the key molecular trigger for biased signaling of GLP-1R as mutations in ECL1, 2 and 3 can convert the receptor preferentially to G-protein or arrestin specific pathways. This is consistent with our observations reported in this paper. The extracellular surface presented by ECL1, 2 and 3 is the binding interface for the GLP-1R ECD, which plays a dominant role in the receptor activation. It is thus reasonable to envision that mutations in the ECLs affect the ECD-TMD interaction, thus affecting the receptor activation conformation and its coupling to specific downstream signaling effectors. Besides the extracellular surface, GLP-1R is likely to have other allosteric sites that regulate ECD-TMD interactions; for example, the positive modulator BETP binds to C347 near to the cytoplasmic side of TM6 [35]. In addition, crystal structures revealed that small molecule antagonists can bind to an allosteric binding site within the TMD bundle of corticotropin releasing factor 1 receptor [14], or to the allosteric site outside the TMD and between TM6 and TM7 of GCGR [15, 16]. Thus, class B GPCRs are allosteric machines that can be regulated by interactions with many sites in the receptor, and these sites could provide tremendous opportunities for drug discovery.

Finally, we found previous reports in support of an ‘endogenous agonist mechanism’ for several class B GPCRs, including the secretin receptor and GLP-1R, in which short peptide motifs from the receptor ECD region can serve as receptor activator [50, 51]. It is very intriguing that the secretin receptor ‘WDN’ motif and and GLP-1R ‘NRTFD’ motif found in the receptor ECD can activate several class B GPCRs to similar maximum levels as their canonical peptide ligands. The ‘endogenous agonist mechanism’ as proposed previously is consistent with the intrinsic agonist model we present in this paper, in which we propose that the ECD is the major determining factor in activation of GLP-1R. The molecular receptor activation mechanisms of these endogenous agonist peptides are currently unknown but the peptides have been cross-linked to the ECL3 region of the receptor [52, 53]. The detailed interaction of these endogenous agonist peptides with the receptor and their interplay with their canonical peptide ligands remain a subject of future studies that awaits the structure elucidation of full-length GLP-1R bound to GLP-1 or EX4.

## Materials and Methods

### Reagents

DNA polymerase and restriction enzymes were purchased from Thermo Fisher Scientific. Dulbecco's modified eagle medium (DMEM)/high glucose and fetal bovine serum used to culture the cells were from HyClone and Gibco. Trypsin-EDTA and PBS were from Gibco. GLP-1(7–37), EX4(1–39) and GLP-1(9–37) for cAMP and tango assays were custom-synthesized and high-performance liquid chromatography-purified by Pepmic, and the EX4(3–39) peptides were purchased from Novopro. Peptides were dissolved in water and diluted to the concentrations required for the assays.

### Mutagenesis

Site-directed mutagenesis was carried out using the QuikChange kit (Agilent, Santa Clara, CA, USA). Mutations and all plasmid constructs were confirmed by DNA sequencing before use.

### Cell culture

The AD293 cell line was cultured in DMEM/high glucose medium with l-glutamine (Hyclone, Logan, UT, USA) supplemented with 10% fetal bovine serum (Gibco, Carlsbad, CA, USA). Cells were grown at 37 °C in a humidified chamber supplied with 5% CO_2_. Cells reaching 80–90% confluence were detached by trypsinization and re-seeded by 4–6-fold dilution in fresh medium in 24-well plates for the assays. The HTL cells were grown in the same condition as the AD293 cells.

### Transfections for cAMP luciferase reporter assays

Codon-optimized cDNAs of human GLP-1R (residues 24–429) and human GCGR (residues 26–431) lacking the predicted signal peptide coding region were synthesized by Genewiz. The receptor constructs were fused with a human IgG leader (MGWSCIILFLVATATGVHSE) at their N-termini for cell membrane localization, and a 3xFLAG tag (DYKDDDDKDYKDDD-DKDYKDDDDKV) at their C-termini for immunoblotting. The fusion proteins were expressed in AD293 cells. EX4(1–39), GLP-1(7–37) and various truncated versions were fused to full-length GLP-1R with a 5xGSA linker. Seamless cloning was used to insert the coding regions into the vector without the use of restriction sites.

AD293 cells were plated at a density of 5x10^4^ per well in 24-well plates 1 day before transfection. Cells were transiently co-transfected with GPCR expression plasmid (50 ng) and the luciferase reporter constructs with cAMP response element (CRE, 200 ng) and TK -Renilla (10 ng) using Lipofectamine 2000 reagent (Life Technologies, Carlsbad, CA, USA) at a ratio of 2:1 (reagent:DNA). Peptides were added to the cells to a final concentration of 1 μm 4 h before collection. All experiments were performed as three independent transfection experiments.

### Luciferase assays

All cells were harvested 24 h after transfection, and lysed in 100 μl Passive Lysis Buffer (Promega, Fitchburg, WI, USA). The cAMP concentration in lysate was determined using the Dual-luciferase reporter assay system from Promega according to the manufacturer’s instructions with an EnVision plate reader (PerkinElmer, Waltham, MA, USA). Renilla luciferase was used for normalization.

### Cell-based assay for detecting the EC50 of peptide hormones

The activation of GLP-1R induced by synthetic peptide hormones including GLP-1(7–37), EX4(1–39), GLP-1(9–37) and EX4(3–39) was evaluated using special AD293 cells that stably expressed human GLP-1R and NanoLuc luciferase under control of a cAMP response element (CRE; pNL-NlucP-CRE-Hygro, Promega). The stable cell line (GLP-1R/NlucP-293 cells) was established by G418 and hygromycin antibiotic selection. GLP-1R/NlucP-293 cells (<15 passages) were plated at a density of 10^4^ per well in 96-well white plates (Corning). A series of peptide concentrations ranging from 30 μm to 0.1 pm in DMEM was added to the cells 4 h before collection. All experiments were performed with three independent treatments of peptides. The cAMP signal of GLP-1R/NlucP-293 cells was determined using the Nano-Glo Luciferase Assay System (Promega) according to the manufacturer’s instructions. The EC50 values (50% maximal effective concentration) were calculated using GraphPad Prism software version 5.0 (La Jolla, CA, USA).

### Cell-based assay for determining the EC50 for the activation of WT and C347K/R mutant GLP-1R by EX4

The activation of WT and C347K/R GLP-1R by synthetic EX4(1–39) was evaluated using AD293 cells or HTL cells. Transfection was done following the same protocol as forhe cAMP-activated luciferase reporter assays. A series of EX4 peptide concentrations ranging from 2 μm to 0.1 pm in DMEM was added to the cells 4 h before collection. All experiments were performed by three independent transfections.

### Cell-based assays for detecting arrestin pathway signaling (Tango assays)

pcDNA6-based fusion constructs were generated by seamless cloning. The receptor constructs consisted of human IgG leader (MGWSCIILFLVATATGVHSE), GLP-1R (residues 24–429), a TEV protease cleavage site (TEV site) and the transcriptional activator tTA (class B GPCR–TEV site–tTA). The ß-arrestin 1 construct encoded ß-arrestin 1 fused with TEV protease (ß-Arr1–TEV protease). The full-length GLP-1 or EX4 peptides or their truncated variants were fused to the N terminus of the receptor with a 5xGSA linker. The HTL cells were seeded in 24-well plates (10, 000 cells per well). When cells reached 15–20% confluence, 10 ng of GLP-1R–TEV site–tTA plasmid were co-transfected with 10 ng β-Arr1–TEV protease plasmid and 5 ng of phRG-tk Renilla luciferase expression vector using X-tremegene (Roche, Indianapolis, IN, USA) at a ratio of 3:1 (reagent:DNA). 24 h after transfection, cells were incubated with 1 μm GLP-1 or EX4 peptide. Cells were harvested after another 24 h and lysed in Passive Lysis Buffer (Promega). Luciferase assays were performed as above.

### Expression of membrane-tethered ECD-peptide hormone fusions

The constructs encoding membrane-tethered peptide hormone that were N-terminally fused with the receptor ECD were generated in pcDNA6. They contain an IgG leader (MGWSCIILFLVATATGVHSE), followed by the peptide hormone, a 5xGSA- linker, the receptor ECDs, an 5xNG linker, a MYC tag (EQKLISEEDL), a G plus 9xNG linker, a single-pass TMD (ALCWVGIGIGVLAAGVLVVTAIVYVV), and a 3X-FLAG tag, as described in previously published literature [36]. The GLP-1R ECD(24-126) and GCGR ECD(26-122) were codon-optimized. The corresponding WT or C347K mutated GLP-1R TMDs (residues 127–429) were cloned with IgG leader at the N terminus and a 3xFLAG tag at the C terminus into pcDNA6. When AD293 cells reached 70–80% confluence, 50 ng GLP-1R TMD (WT or C347K) plasmid were co-transfected with 50 ng membrane-tethered ECD-peptide/ECD fusion plasmid, 200 ng of the CRE-luciferase reporter construct and 10 ng of the TK–Renilla construct using Lipofectamine 2000 reagent (1:2).

### Co-expression of membrane-tethered EX4 and free EX4 peptide hormone

Codon-optimized sequences encoding EX4 peptide hormone were inserted in frame into the vector expressing 5xNG linker followed by a MYC tag, a G plus 9xNG linker, a single-pass TMD, and a 3X-FLAG tag. Non-fused EX4 was expressed with an introduced TAA stop codon at the end of the EX4 peptide. When AD293 cells reached 70–80% confluency, 50 ng full-length GLP-1R (WT) plasmid were co-transfected with 50 ng membrane-tethered-peptide plasmid or 50 ng plasmid expressing free (non-membrane tethered) EX4. Luciferase assays were performed as above.

### Western blots

AD293 cells were split 1 day before transfection at 10^6^ cells per well in a 12-well plate. Cells were grown for 1 day, then transfected with 1 μg GLP-1R construct (pcDNA6-GLP-1R-3xFLAG) using Lipofectamine 2000 (DNA/Lipofectamine 2000 ratio of 1:2) in each well. One day after transfection, cells were harvested by centrifugation and their pellets were solubilized in cell lysis reagent (Cellytic M, Sigma, St Louis, MO, USA) supplemented with 1 mm PMSF and centrifuged at 16 000 *g* for 30 min. The supernatants were subjected to reducing sodium dodecyl sulfate-polyacrylamide gel electrophoresis and transferred to polyvinylidene fluoride membranes. The membranes were blocked with 5% milk in TBST (20 mm Tris-HCl pH8.0, 150 mm NaCl and 0.05% Tween-20) for 1.5 h, then incubated with horseradish peroxidase-conjugated anti-Flag (Sigma M2) antibody or monoclonal anti-β-actin antibody produced in mouse clone AC-15 (Sigma).

### Statistical analysis

GraphPad Prism software version 5.0 (GraphPad Software Inc, San Diego, CA, USA) was used to fit data to a three-parameter dose-response curve. The bar graph data are presented as mean±s.d., and curve data are presented as mean±s.e.m. The statistical significance of all data reported in this paper was determined with Student’s *t* test analyses. Western blot signal intensities were quantified by integrating the luminosity curve of selected lanes using ImageJ [54]. The relative expression was calculated using the (target band signal intensities)/(corresponing β-Actin signal intensities) relative to WT control.
