# Supplementary material for: An intrinsic agonist mechanism for activation of glucagon-like peptide-1 receptor by its extracellular domain
Source: Cell Discov. 2016 Nov 22;2:16042–. doi: 10.1038/celldisc.2016.42 (PMC5118412; doi:10.1038/celldisc.2016.42)

Supplementary Figures

An Intrinsic Agonist Mechanism for Activation of Glucagon-Like Peptide-1 Receptor by its Extracellular Domain

Yanting Yin[1,2,3], X. Edward Zhou[2], Li Hou[1], Li-Hua Zhao[1], Bo Liu[1], Gaihong Wang[1], Yi Jiang[1], Karsten Melcher[2], H. Eric Xu[1,2,3]

[1]Key Laboratory of Receptor Research, VARI-SIMM Center, Center for Structure and Function of Drug Targets, Shanghai Institute of Materia Medica, Chinese Academy of Sciences, Shanghai 201203, China.

[2]Laboratory of Structural Sciences and Laboratory of Structural Biology and Biochemistry, Van Andel Research Institute, 333 Bostwick Avenue Northeast, Grand Rapids, MI 49503, USA.

[3]University of Chinese Academy of Sciences，No.19A Yuquan Road, Beijing 100049, China.

Correspondence: Karsten Melcher, Karsten.melcher@vai.org or
H. Eric Xu, eric.xu@vai.org

Supplementary Figures

Figure S1. GLP-1R expression levels

Immunoblots showing **(A)** The expression levels of wild type GLP-1R fused with truncated EX4 peptide hormone. **(B)** The expression levels of wild type or C347K/R mutated EX4(8-39)-5GSA-FL constructs. **(C)** The expression levels of truncated EX4 peptide fused to the full length and C347K mutated GLP-1R. All Immunoblots were performed with anti-FLAG antibody for detection of receptor expression and β-actin antibody for normalization. Relative expression of the receptor with the truncated peptide fusions are against the expression of the receptor fused with the full length EX4 peptide (Δ0, marked with red asterisks). G: glycosylated receptor, N: non-glycosylated receptor. RG: relative expression of glycosylated receptor, R: relative expression of non-glycosylated receptor. RT: relative expression of total receptor (glycosylated and non-glycosylated receptor).

Figure S2. The expression levels of EF-GS(A) linker-fused GLP1R and GLP-1R TMD with alanine replacements in the extracellular loops

(A) The expression levels of linker-fused GLP-1R. **(B-D)** Immunoblots showing the expression levels of GLP-1R TMD with alanine mutation in extracellular loops 1 **(B)**, 2 **(C)**, and 3 **(D)**. All Immunoblots were probed with anti-FLAG antibody for expression detection and β-actin antibody for normalization. Expression levels of the receptors are indicated as relative to the FL WT or TMD, which were set at 1.00, G: glycosylated receptor, N: non-glycosylated receptor. RG: relative expression of glycosylated receptor, R: relative expression of non-glycosylated receptor. RT: relative expression of total receptor (glycosylated and non-glycosylated receptor).

Figure S3. Expression-normalized cAMP signal produced by EX4-ECD-M and GLP-1R TMD with indicated mutations in ECL1 **(A)**, 2 **(B)** and 3 **(C)**. Normalized data correspond to the non-expression normalized data in Figures 8A, 8C and 8E.

Supplementary figure 1

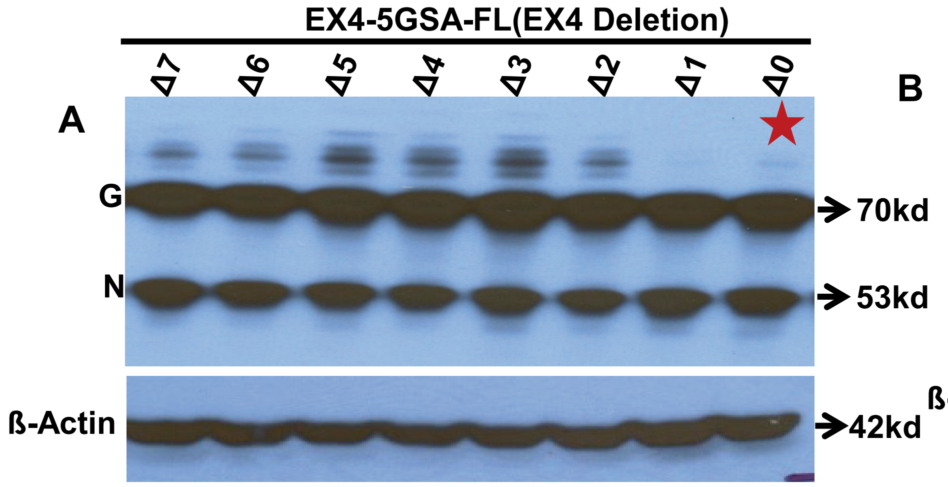

RG	0.91	0.95	1.12	1.13	1.22	1.01	0.93	1.00
RN	0.93	1.01	1.05	0.97	1.03	0.90	0.92	1.00
RT	0.91	0.98	1.09	1.06	1.14	1.01	0.93	1.00

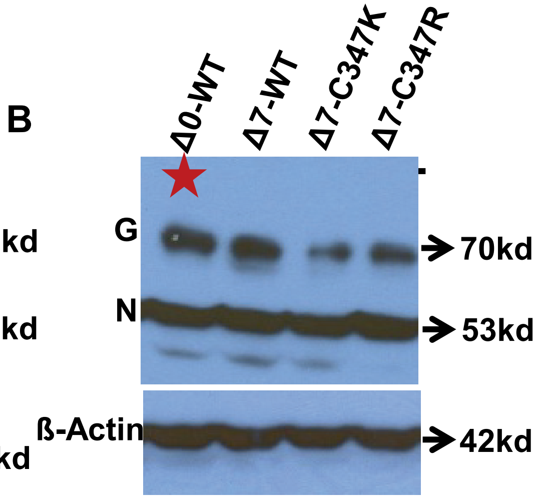

RG	1.00	1.23	0.61	0.87
RN	1.00	1.19	1.21	1.25
RT	1	1.21	0.96	1.09

C

EX4-5GSA-FL (C347K) EX4 Deletion

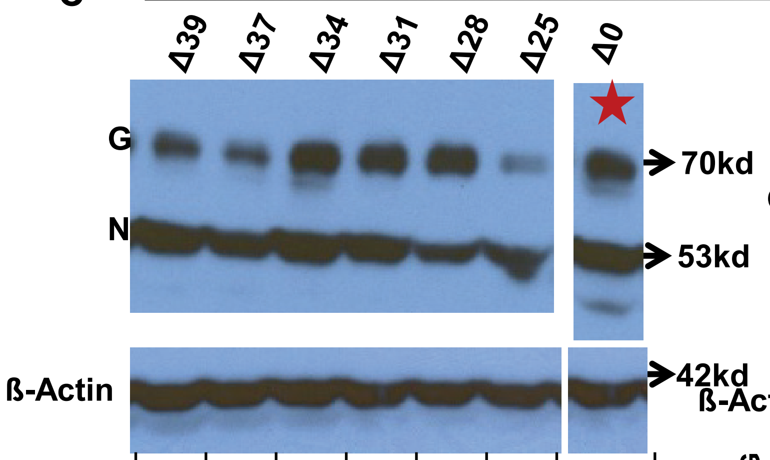

RG	0.73	0.62	1.28	1.14	1.20	0.34	1.00
RN	1.01	0.89	1.03	1.02	0.76	0.95	1.00
RT	0.89	0.77	1.03	1.06	0.94	0.70	1.00

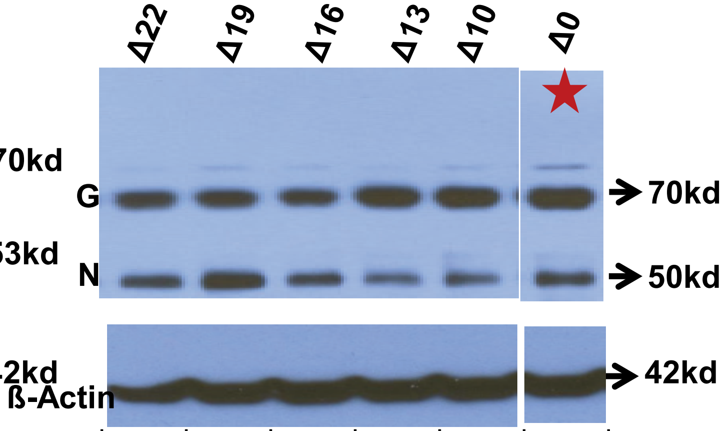

RG	0.91	0.81	0.63	0.89	0.87	1.00
RN	1.17	1.37	0.78	0.62	0.65	1.00
RT	1.00	1.01	0.68	0.79	0.79	1.00

Supplementary figure 2

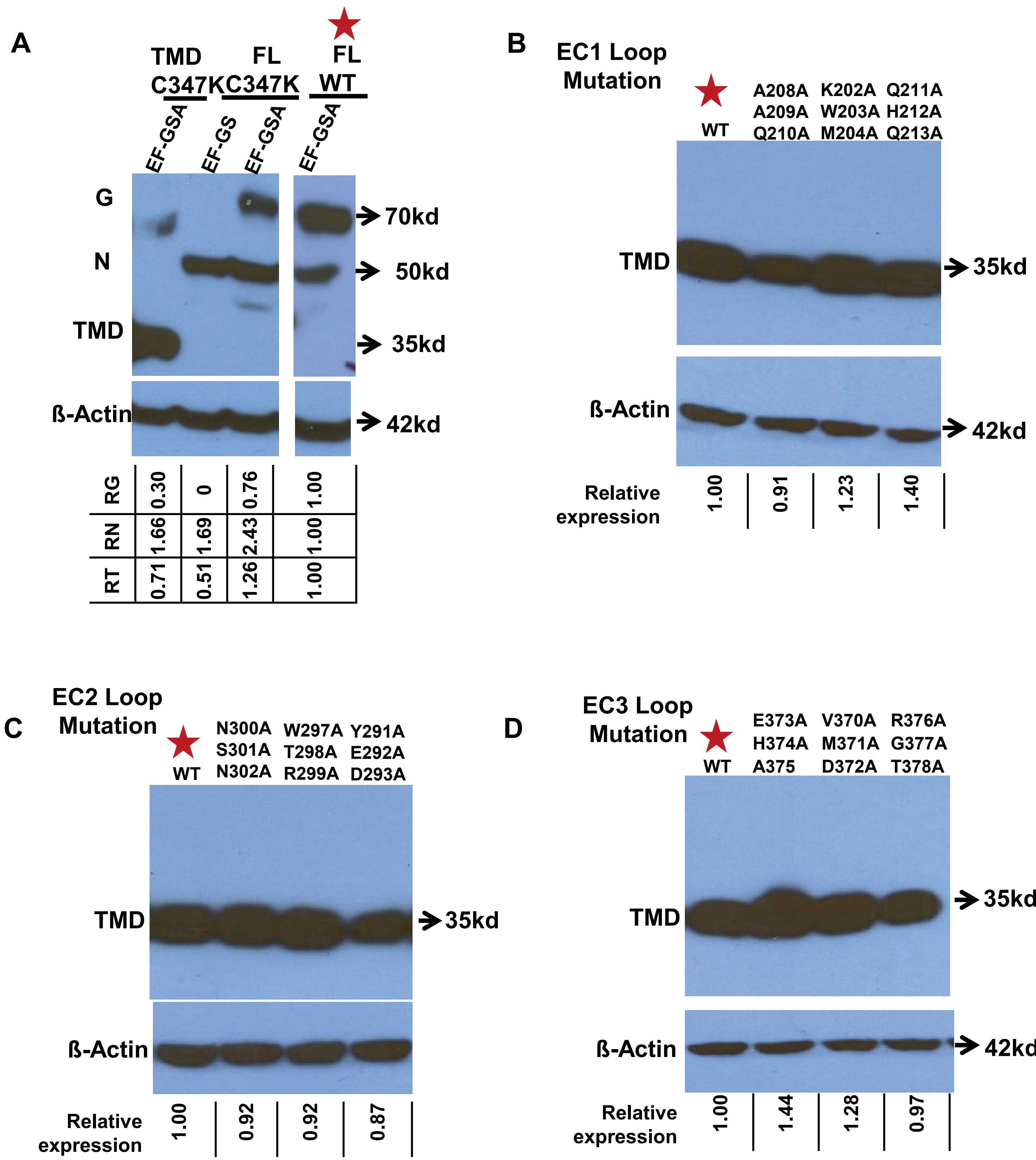

Supplementary figure 3

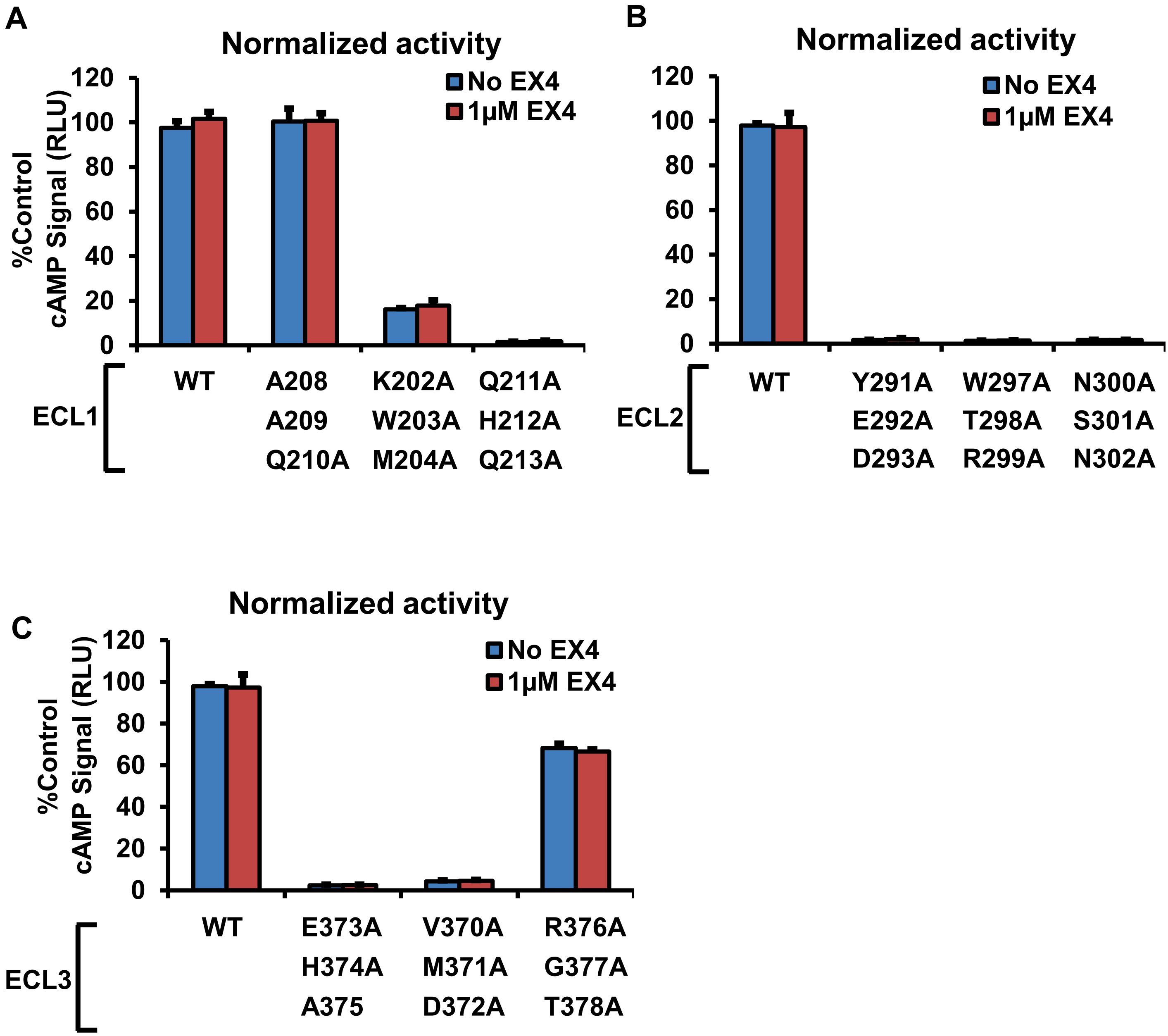

Supplement: Supplementary Information [file celldisc201642-s1.pdf]
